# Supplementary material for: LWLCM: A novel lightweight stream cipher using logistic chaos function and multiplexer for IoT communications
Source: PLoS One. 2025 Sep 4;20(9):e0330976. doi: 10.1371/journal.pone.0330976 (PMC12410779; doi:10.1371/journal.pone.0330976)
Supplement: Attachment — Submitted filename: PONE-D-25-29174.R1-Response-File.docx [file pone.0330976.s002.docx]

**Manuscript title:** **LWLCM: A Novel Lightweight Stream Cipher Using Logistic Chaos Function and Multiplexer for IoT Communications**

**Manuscript ID: PONE-D-25-29174.R1**

The authors would like to thank the editor and the anonymous reviewers for their comments/suggestions received to improve the quality of the paper. As per the direction, the point-wise responses/explanations to all comments from the anonymous reviewers are given, and the corresponding revised portion in the revised manuscript is highlighted in yellow and green.

**Reviewers' Response**

**Comments to the Author
Reviewer #1:**

1. **The chaos function used is only the standard logistic map, without structural modification or combination with other maps or modifications to improve**

**the quality of the chaotic map. The reason for choosing the logistic map needs to be explained because there are many new models that are lighter and more robust. In this regard, the introduction needs to be strengthened by explaining the weaknesses and strengths of several recent studies, such as https://doi.org/10.62411/faith.3048-3719-93 and 10.32604/cmc.2024.058478, so that the reason for choosing logistic in this case is stronger.**

**Response:** Thank you for your valuable suggestion. To address your suggestion, we have revised the Introduction to include a comparative discussion on the strengths and weaknesses of recent chaotic systems as presented in [https://doi.org/10.62411/faith.3048-3719-93] and [10.32604/cmc. 2024.058478].

2. **There is no ablation study that tests the role of each component (chaotic module, NLFSR, MUX) separately. This makes the individual contribution of the chaos function to security enhancements unconfirmable experimentally.**

**Response:** Thank you for your observation. We have now included a detailed ablation study in **Section 6.8 on Page No. 33**, evaluating the individual impact of the chaotic module, NLFSR, and MUX on encryption performance.

3. **The description of the output function that combines bits from registers and chaos is not mathematically formalized. The formula and bitwise structure used in the keystream function should be presented explicitly so that it can be replicated.**

**Response:** Thank you for the valuable feedback. We have updated the manuscript to include a mathematically formalized expression of the output function in **Section 3.1.7 on Page No. 16**, detailing bitwise operations and keystream generation structure for reproducibility.

4. **It is not explained how the chaos parameters (α and x₀) are generated.**

**Response:** We appreciate the comment. In **Section 3.1.1** **on Page No. 13**, we have now clarified that the chaos parameters α and x₀ are deterministically derived from the input key and IV using a lightweight transformation function, ensuring both reproducibility and secure initialization.

5. **It is also not explained how the chaos parameters (α and x₀) are stored or transmitted securely in an IoT communication scenario. This ambiguity leaves a gap for potential parameter recovery attacks.**

**Response:** Thank you for pointing this out. In **Section 3.1.1** **on pages 13-14**, we have now explained that the chaos parameters (α and x₀) are not transmitted separately but are securely regenerated at the receiver’s end using the shared key and IV. This design ensures that the parameters remain confidential and eliminates the risk of exposure to parameter recovery attacks.

6. **There is no clear explanation of the type of plaintext encrypted in the test.**

**Response:** We appreciate the observation. **In Section 6 on Pages No. 22-23**, we have clarified that the plaintexts used in the encryption tests are real-world text-based data files representing system messages and communication logs, ranging from 2^6^ to 2^12^ bytes in size, to simulate typical IoT data payloads. We believe that these revisions enhance the clarity and comprehensiveness of our research.

7. **Then how to do the comparison, is it with a replication technique or how?**

**Response:** Thank you for the comment. The comparison was conducted by implementing replication models of the proposed cipher, each with specific components (chaotic map, NLFSR, and MUX) excluded individually. This ablation study, detailed in **Section 6 on Page No. 22**, quantifies the individual impact of each module on encryption performance.

8. **The avalanche effect test in this paper is carried out on a 1-bit variation in the key, but it is not explicitly explained which part of the key is modified — whether it is the initial seed of the chaos function, the state of the LFSR/NLFSR, or the control bits in the MUX. This ambiguity makes replication and interpretation difficult. In addition, there is no avalanche effect test for a 1-bit change in the plaintext, which is important for evaluating the effects of cipher diffusion in practical scenarios.**

**Response:** Thank you for the comment. In our avalanche effect evaluation, a 1-bit change was applied to the 128-bit secret key, which alters the initial conditions of both the chaotic generator and the NLFSR. We have clarified this aspect in **Section 6.5 on Page No. 28**. As stream ciphers operate on a bit-by-bit keystream XOR mechanism, measuring the avalanche effect due to plaintext changes is not straightforward. Therefore, we have focused our avalanche analysis solely on key sensitivity, which reflects the cipher's robustness against key-related differential attacks.

9. **Explained in the data availability statement "The data set generated during and/or analyzed during the current study is available in the KAGGLE repository,**

**https://www.kaggle.com/datasets/crawford/20-newsgroups" Why choose this dataset, but not**

**explained in section 4 or 3 clearly?**

**Response:** Thank you for the valuable feedback. We have updated **Section 6 on Page No. 22** to clearly explain the rationale for choosing the 20 Newsgroups dataset. This dataset offers a diverse and unstructured text corpus ideal for evaluating the encryption performance on real-world plaintext. It enables the assessment of statistical and security metrics under various textual patterns, making it suitable for validating the proposed lightweight stream cipher.

**Reviewer #2:**

1. **The statement in the manuscript that “LWLCM is the first time anyone has tried to adapt chaotic structures to lightweight stream ciphers” is overly absolute. In fact, previous studies have combined chaotic systems with nonlinear feedback shift registers to design stream ciphers. This statement does not adequately consider the existing technical background.**

**Response:** We appreciate the reviewer’s observation. The statement has been revised in **Section 1.2 on Page No. 5** to avoid absolutism. We now acknowledge previous efforts combining chaotic systems with NLFSRs in stream cipher design and emphasize that our novelty lies in the specific integration of the logistic map, dynamic MUX control, and lightweight architecture tailored for constrained IoT devices.

2. **The literature lacks currency, with several references older than five years. We recommend updating the manuscript with recent work to reflect current research progress**

**Response:** Thank you for the insightful comment. We have updated the literature by incorporating several recent studies to reflect the current state of research and enhance the manuscript’s relevance and rigor

3. **Section 1.1 points out that classical cryptographic algorithms are not applicable to restricted devices due to resource consumption, but fails to explain Why other lightweight classes such as lightweight block ciphers and hash derivation schemes have limitations of applicability, and instead discusses stream ciphers directly.**

**Response:** Thank you for your observation. In the revised manuscript in **Section 1.1 on Page No. 4**, we have included a comparative discussion highlighting the limitations of lightweight block ciphers and hash derivation schemes—such as higher latency, limited adaptability for continuous data encryption, and increased hardware complexity—thereby justifying the focus on stream ciphers for real-time, resource-constrained IoT environments.

4. **The logic of the contribution (1.2) and page 6 section is unclear and does not highlight the core innovations. The chaotic pseudo-random number generation module mentioned in the abstract is not clearly explained in the contribution section, and the description of the dynamic adjustment mechanism of the multiplexer lacks technical details (such as control logic)**

**Response:** Thank you for your valuable feedback. We have revised **Section 1.2** to clearly highlight the core innovations of the proposed LWLCM scheme. Specifically, we now elaborate on the chaotic pseudo-random number generation module, detailing its integration with the LFSR and NLFSR. Additionally, we have clarified the dynamic adjustment mechanism of the multiplexer, including the control logic based on selected bits from the LFSR, which introduces additional non-linearity and enhances resistance to structural attacks.

5. **Some custom symbols and formula symbols in this manuscript are not explained in detail, such as the symbols in Equation 1.**

**Response:** Thank you for pointing this out. We have updated the manuscript to provide detailed explanations for all custom and mathematical symbols used, especially those in Equation 1. Each symbol is now clearly defined either directly below the equation or in a dedicated nomenclature subsection, ensuring clarity and ease of understanding for the readers.

In section 5. "Implementation" on **Page No. 19**, we have provided detailed descriptions of the design and implementation of our algorithm.

6. **The manuscript's section 7 “Security Analysis,” lacks experimental data and comparative verification. It lacks test data for attacks such as linear approximation attack and correlation attack, and does not compare security metrics with other algorithms. As a result, the claim of resistance to various attacks lacks quantitative support.**

**Response:** We appreciate the reviewer’s observation. In response, we have revised **Section 7 on Pages No. 35-36** to include experimental evaluations for resistance against linear approximation and correlation attacks.

7. **The statistical testing section in Chapter 8 of the manuscript has issues with test completeness. Although the paper mentions that LWLCM passed all 16 NIST tests and Shannon entropy is close to the theoretical maximum value, it does not provide detailed information on test sample length, number of repetitions, and other parameters.**

**Response:** Thank you for highlighting this point. We have updated **section 8 on Page No. 39** to include detailed information regarding the statistical testing setup. Specifically, the NIST test suite was conducted on a 1,500,000-bit keystream, and each test was repeated 100 times to ensure statistical consistency. The average P-values for all 16 tests are now included in a summary table, along with parameters such as sample length and confidence thresholds, enhancing the reproducibility and transparency of our results.

**With Best Regards**

**Zubair Ashraf:** ashrafzubair786@gmail.com & **Mahfooz Alam:** [mahfoozalam.amu@gmail.com](mailto:mahfoozalam.amu@gmail.com)

(On Behalf of all authors)
